# A Case of Intraorbital Hemangioma Diagnosed with Tc 99m Labeled Erythrocyte Scintigraphy and Magnetic Resonance Imaging

**DOI:** 10.4274/MIRT.20.05

**Published:** 2011-04-01

**Authors:** Tansel Ansal Balcı, Zeyra Pınar Koc, Burak Turgut, Ayse Murat Aydın, Bedriye Busra Demirel

**Affiliations:** 1 Fırat University Medical Faculty, Departments of Nuclear Medicine, Elazig, Turkey; 2 Fırat University Medical Faculty, Departments of Nuclear Medicine Ophtalmology, Elazig, Turkey; 3 Fırat University Medical Faculty, Departments of Nuclear Medicine Radiology, Elazig, Turkey

**Keywords:** Hemangioma, Technetium Tc 99m pyrophosphate, orbital neoplasms, Radionuclide imaging, magnetic resonance imaging

## Abstract

We present a 20 year-old male patient who had prominent positional proptosis of the right eye and admitted to the hospital for tonsillectomy operation. After conventional ophthalmological examination, magnetic resonance imaging (MRI) and Tc 99m labeled erythrocyte scintigraphy were performed to confirm the diagnosis. Although it is rare to perform scintigraphy for this pathology, the visualization of intraorbital hemangioma was very obvious and we would like to present the visualization of the intraorbital hemangioma both with scintigraphy and MRI.

**Conflict of interest:**None declared.

## INTRODUCTION

The most common benign orbital tumors of the childhood are hemangiomas (1). Capillary hemangioma is a benign vascular lesion that is usually not apparent at birth, but it proliferates during the first year of life and regresses about age seven (2). Periorbital hemangiomas are usually involute and cause no ocular pathology (3). However, amblyopia is the most frequent complication. The others are strabismus, ptosis, proptosis, exposure keratopathy and optic atrophy (4).

Our case presented with proptosis, especially during the hyperflexion of the neck. Scintigraphic imaging of orbital tumors is a rare approach during the diagnostic course. However, in some previously reported cases, Tc 99m labeled erythrocyte imaging was used and the diagnosis was confirmed with histopathological results (5). Differentiation of the hemangioma from vascular malignant tumors might be difficult by MRI for some cases but MRI can identify the lesion borders, nature and relationship with the adjacent soft tissue structures. Confirmation of the diagnosis of these tumors by Tc 99m labeled erythrocyte imaging which clearly demonstrates hepatic hemangiomas, will be better if the MRI results are suspicious. We present an intraorbital hemangioma case imaged with Tc 99m labeled erythrocyte scintigraphy and MRI. 

## CASE REPORTS

A right sided proptosis in a 20 year-old male patient who underwent tonsillectomy operation was evaluated. He declared that this pathology had existed since his childhood. Ophthalmological examination, MRI and labeled erythrocyte scintigraphy were performed. On ophthalmological examination, mild exophthalmus was present during the hyperflexion of the neck. Visual acuities were 20/20 on both eyes. Although Hertel exophthalmometry could not be performed, significant proptosis was visually observed on his right eye during the hyperflexion position. Hertel exophthalmometry on primary position measured 11.6 mm and 11.2 mm for the right and left eye, respectively. Eye movements were intact. 

**Figure 1:**
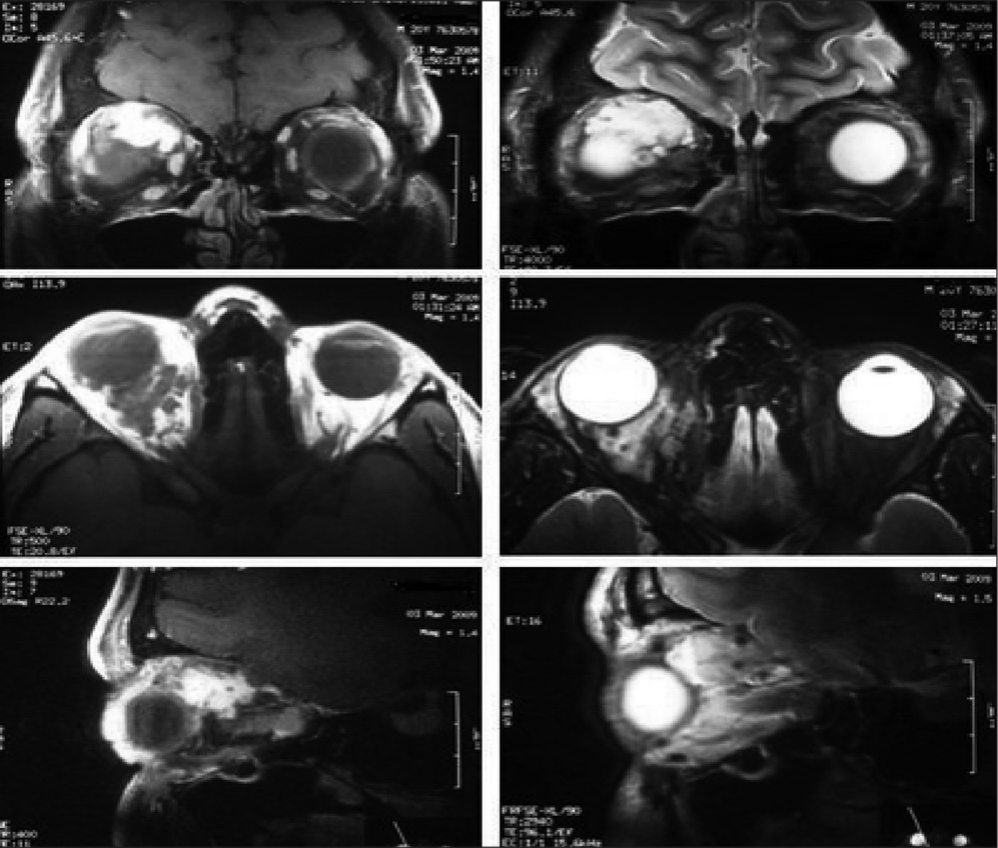
Coronal (T1-w contrast, T2 w/out contrast), axial (T1 and T2), and sagittal (T1 and T2) slices of MRI

**Figure 2:**
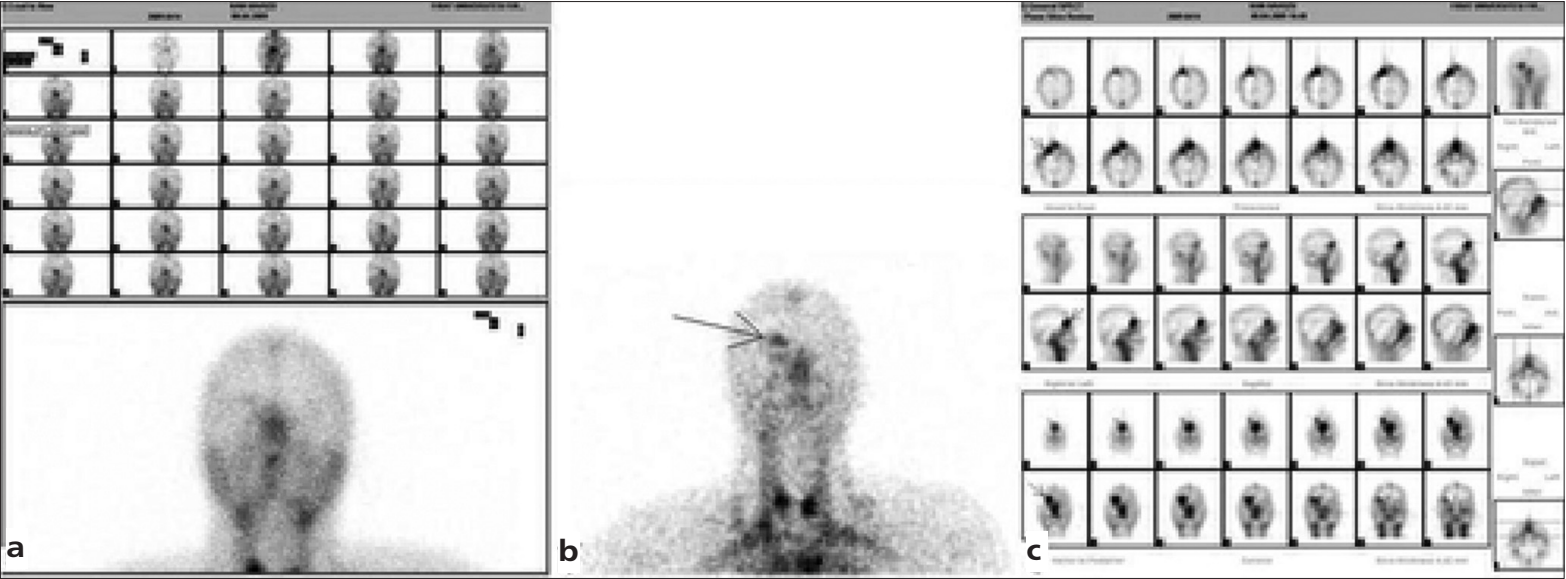
Dynamic (serial images on the first rows of 2a), early blood-pool (bottom image of 2a), late phase planar (2b) images on the anterior projection and SPECT (2c-transaxial, sagittal and coronal slices, respectively) images of Tc 99m labeled erythrocyte scintigraphy

Slit-lamp biomicroscopy and fundus examination did not show any pathological findings. There is no family history. MRI (Figure 1) showed minimal exophthalmus and the lesion which is 3x3 cm in size. The lesion at the right intraconal localization and causing minimal depletion of the superior rectus muscle showed lobulated borders with signal void areas, isointense on T1-weighted and hyperintense on T2-weighted images. After intravenous contrast injection; the lesion showed intense contrast enhancement. We performed in vivo labeled Tc 99m erythrocyte scintigraphy. Dynamic images showed no vascularisation, and there was slightly increased activity on the early blood-pool images (Figure 2a). Delayed phase planar and SPECT images showed intense tracer accumulation on the lesion localization (Figure 2b, 2c). This typical scintigraphic pattern is known as “perfusion-blood pool mismatch”. 

## DISCUSSION

The diagnostic test of choice is MRI for intraorbital hemangioma (6) which is characterized by indefinite borders usually penetrating into adjacent tissues. The diagnostic hallmark of the image is numerous vascular structure content of the lesion (7). Tc 99m labeled erythrocyte imaging for hemangioma is usually used for the diagnosis of liver hemangiomas. Although there is a significant physiological background tracer accumulation, diagnostic performance of this method for liver lesions is very precise. This technique can also easily demonstrate this kind of tumors even if they are in unusual localization as there is little background activity. Radioactivity accumulation within the lesion of our patient was very prominent in the late phase images. The scintigraphic pattern of the hemangioma which is hypoactive in the vascular phase and hyperactive in the late phase, is previously well established (8,9,10). In some studies, this typical appearance of these tumors was used for the differentiation from malignant tumors and confirmed with histopathology (5). According to previous reports, Tc 99m labeled erythrocyte imaging could differentiate hemangioma from malignant tumors (5,8,9,10). This characteristic of Tc 99m labeled erythrocyte scintigraphy might help to decide the choice of treatment, i.e. surgery for the malignant lesions or follow-up for the hemangiomas. Finally, appropriate management for hemangiomas is to follow-up by means of clinical and radiological findings and intralesional corticosteroid injection 10 or surgical intervention in some rare situations (11).
